# Supplementary material for: Pre-analytical errors during HIV viral load (HIV-VL) and early infant diagnosis (EID) testing in Cameroon
Source: PLoS One. 2026 Apr 27;21(4):e0324886. doi: 10.1371/journal.pone.0324886 (PMC13120008; doi:10.1371/journal.pone.0324886)
Supplement: Attachment — Submitted filename: Review Comments_VLEID_PLOS One.pdf [file pone.0324886.s006.pdf]

Identification of common pre-analytical errors during the HIV viral load and EID testing in Cameroon: A call for strengthening the laboratory systems to support follow-up people living with HIV/AIDS.

Review Comments – Dr. Nanyeenya Nicholus

A. General Comments

This is a very critical study which seeks to provide more knowledge about pre-analytical errors in Viral load (VL) and EID testing in Cameroon in West Africa. Both VL and EID in West Africa are being scaled up, and facing different challenges like increased sample rejection due to pre-analytical errors. Hence such a study will produce key information that might inform key policy decisions in the entire region. However the study still need significant improvements in all the sections. The introduction needs to be revised to set the scene for the study. The methods are grossly under-reported and need to be overhauled with a comprehensive data analysis, with support from a good Biostatistician to bring out the study results well. Consequently, the results section also needs an overhaul, re-writing the section to address the different comments below.

B. Specific Comments

1. Introduction: The authors have written a fairly good and concise introduction. However there are a number of issues that need to be rectified, to make the introduction better and these include;
   a) The introduction does not set the scene for the study. Since the study is about pre-analytical errors for HIV VL and EID, there is need to set up the scene, starting with a brief description of the i) burden of HIV in Cameroon, ii) progress made in treatment coverage, iii) progress made in laboratory with a focus on diagnosis in infants (EID) and monitoring of ART (VL), hence this introduces the concepts of VL and EID, iv) the challenges faced, including pre-analytical errors, and this can then be described comprehensively, and then v) how the study is important. The study introduction does not chronologically flow well, and misses linkage of all these aspects. Revise the introduction to include all these aspects.
   b) The literature citation in the introduction is also sub-optimal. There is need to do a thorough literature review around the pre-analytical errors in VL and EID testing and use them to build a case here.
   c) A brief description of the VL and EID testing services and their coverage would also strengthen the introduction.
   d) This is an important topic, but the authors do not elaborate well about the problem in this study, and actually the actual problem being addressed remains slightly unclear.
      i) Hence there is need to clearly define the problem in this study, and its magnitude
      ii) What has been done before about the problem and the risk of not addressing the problem? This should all be clear in the introduction.

2. <u>Methods:</u> The methods section is generally very sub-optimal, with several issues to address;
   a) The sampling and data collection section is very unclear. How was the sampling done in each facility? What was the sample size? Were all the eligible samples assessed? What was the inclusion and exclusion criteria for the samples? How was the semi-administered questionnaires developed, piloted and used? Which key variables were collected?
   b) The section of program description (how the programs run) could be removed from the section of sampling and data collection and made to stand alone. This will make the methods section better.
   c) The statistical analysis section is also missing the key analysis techniques that were used, which makes it very hard to understand how the data was analysed. Was there missing data? And if yes, how was it managed?
   d) For the ethical consideration, I am not sure whether it was right not to review the study simply because it was retrospective. I would rather think that the study would have been reviewed by the Ethics Committee but instead the informed consent would have been waived given its retrospective nature. You will kindly need to clarify this, as it might raise ethical concerns.

3. <u>Results:</u> In the results section, the descriptive statistics are sub-optimally presented. Including a table with key variables like age and gender is key for the results section.
4. The data analyses also miss detailed analysis to understand how key variables like age and gender among others affect sample rejection.
5. Both tables 1 and 2 are not clearly drawn and they are very difficult to follow-through and understand. For table 1, normally the frequency/number is together with the percentage in brackets for example n (%) i.e 10508 (5.6). Kindly re-draw these tables so that they are easy to understand.
6. I generally feel the authors need to work with a good Biostatistician and re-analyze the results well using good statistical techniques, and also re-write the entire results section to improve it.
