# Supplementary material for: Pre-analytical errors during HIV viral load (HIV-VL) and early infant diagnosis (EID) testing in Cameroon
Source: PLoS One. 2026 Apr 27;21(4):e0324886. doi: 10.1371/journal.pone.0324886 (PMC13120008; doi:10.1371/journal.pone.0324886)
Supplement: Attachment — Submitted filename: AM reVIEW NOTES .docx [file pone.0324886.s005.docx]

Thank you for the opportunity to review the manuscript titled *Identification of common pre-analytical errors during the HIV viral load and EID testingin Cameroon: A call for strengthening the laboratory systems to support follow-up people living with HIV/AIDS*.. This paper addresses a rather complex and important aspect which is a strategic entry point for improving diagnostic quality, strengthening laboratory systems, and protecting patient outcomes. It turns operational data into actionable insights that help both patients and health systems function more effectively.

While a useful manuscript, I felt there was room for improved clarity around the methodology used, and the analysis done in order to share best practices of analysing this important issue with other countries

**General /Major comments**

There seemed to be a lot of great work behind the paper covering the spectrum of pre- analytical issues, however, it was hard to follow at times as a new reader on the chronology of the approaches to data collection and analysis.

Some of the methods read more like results, and some of the results read more like conclusions. Recommend re-organizing to make it easier for readers. It would be really helpful to have a summary table of the …..

The statistical analysis could be enhanced to include missing elements on descriptive, and inferential analysis – for example in addition to the Frequencies and proportions of rejected samples (overall and by lab/test type) and Rejection rates (%), and the Breakdown by rejection reasons (e.g., hemolysis, clotted samples, insufficient volume, wrong container, etc.), Stratifying by: Laboratory level (if applicable), Region/district, Facility type (public vs. private), Time (monthly/quarterly trends) could make the findings more insightful

The results section could be better structured in terms of flow – start with the results from descriptive then move to inferential analysis- and may be use trend analysis to assess whether rejection rates are improving or worsening over the 12 months

At present the elements seems mixed – for example table 1 – there is reference to significant correlation of number of labs to number of samples – the percentages in the text are not easily seen how they are derived in the table.

Within the results section Can the authors present results only without interpretation,

In discussion some elements presented in findings are critiqued for example the analysis of samples collected within same region vs those outside

**Minor Comments**

**Background**

**Line 85** – “ *increasing healthcare costs*” – It may be helpful fpr the reader if the authors qualify how high rejection rates increase costs – and not assume its obvious the reason for costs

Be Consistent use of terminology e.g sample and specimen are interchanged in many places

**Line 113** – Validate line 113- may be good to indicate what specific knowledge gap

Is it Gap in magnitude of the problem elsewhere

The rationale for rationale for analyzing rejection is not coming out clearly ? What is the gap the study is closing – the first time for such large-scale assessments in country – this statement is in discussion and may need to be brought up here?

**Methods**

- It is hard to follow the step by step process taken by the authors
- The questionnaire – it will add value the authors add what sort of variables were collected.
- **Line 144- 148** - the description here is of what happens in the current situation and does not part come out clearly how this is part of the methodology used in this study
- **Line 152** – ID given to two different patients – this is not very clear as a reason for rejection – is it labID – Aren’t there other forms of id that complement the unique ID ? etc
- Figure 1 key

Line 155- *The description of Reasons for sample rejection in reference laboratories sounds like results*

*Data Management and analysis* The authors need to articulate how data was management from collection , storage, cleaning analyzed – was it all quantitative data?

Overall, Method needs a more of the above elements to ensure it’s clear

**Results**

- See notes on analysis – and the presentation could follow the same format
- Present results without interpretation
- Mix up between region and ref labs - clarify assumptions- is there no referral of samples - is region from the forms or the lab ?

**Analysis**

There are some gaps specific in terms of what statistical analysis were done

**Eg**

*Descriptive Analysis.* In addition to the summary the data:

- Frequencies and proportions of rejected samples (overall and by lab/test type). It may add value add the totals and show the overall rejection rate. More insight to also Stratify by:
  - Laboratory level – are all these at the same level - all national ?
  - Region/district
  - Facility type (public vs. private)- are they all public ?

Inferential Analysis

*Table 2 does some May be could test hypotheses or relationships – is there a way of including the p values on the table as well- some are mentioned in text which is ok but the table should be strong enough to stand alone*

Using  Chi-square test

- For association between categorical variables (e.g., type of rejection vs. lab level, or region).
- Example: Is the rejection reason associated with the type of health facility?

*Or* **Logistic Regression**

- *You may want to* ***identify predictors of sample rejection*** *(binary outcome: rejected = 1, accepted = 0). Include independent variables like:*
  - *Sample type (plasma, DBS)*
  - *Collection site*
  - *The trained Personnel stated (phlebotomist vs. nurse)*
  - *Transport time- this is probably the samples collected in same region ?*
  - *Facility level*

***Outcome****: Odds Ratios (OR) to interpret influence of variables.*

*And ,* ***trend analysis***

- *Since data was collected over 12 months* – data was collected frp. *11/01/2024 to 08/12/2024* once do we have data showing rejections over time . IF so

*,* ***trend analysis***  (monthly/quarterly trends) *to assess whether rejection rates are improving or worsening can be a good consideration . A line graph, OR  seasonal decomposition could show better insights – may be in a certain season there are issues of sample transport etc*

- I found it difficult to follow the overall N in Table 2 when it compares the association between categorical variables (e.g., type of rejection vs. location of lab within region). Its not clear which one of the two Row percent **or** column percent adds to 100%. Also found it difficult the relationship to Table 1 especially without the N in the title of the tables
  - It will be great to specify the types of EID samples – there is reference to clotted samples – was this whole blood tested at near POC ? Was there a difference in rejection when compared to DBS cards
  - Similarly what type of samples were used for Viral Load ? PSC, EDTA whole blood or PS tubes etc
- It will also be insightful to show association with other variables e.g those stated under inferential statistics above

Line 193-194 – this sound like a method and not result

195-199- consider using a clearer statement **–** define rejection rate in methodology then use statements like overall rejection rate was 3.9%. The rejection rate for HIV VL was x % (range) and for EID was y%.

Need to be clear on primary analysis and secondary analysis

Line 222-228 – the outcomes of the further evaluation stated here made reference to table 2 but this is not shown in table 2

Line 225 – The meaning of “*non-permanent marker identification*” is not always clear to everyone```’ – this may need to be clarified

Table 3 makes reference to collection capacity but it is not clear what collection capacity means? it will be helplful to ensure this is defined – this variable can be in table 2

Both table 2 and 3 could be improved to include the data by region -

**Discussion**

- Line 250 -254 - This first paragraph comes in without context - I would prefer that t the first statement address the primary objective or finding which is “ *The overall rejection rate of specimens among participating labs Was high almost double the expected set limit* – then move on to expand this was seen for both types of tests and by locations
- Some of the elements in the results have not been discussed e.g the relevance of In health facilities with high capacities for sample collection vs the rejection rates , or same region vs other region – is this a proxy for sample transport distance ?

**Other considerations for the authors**  - do we know the cost of each test collection - this could be translated to resources wasted and can be a powerful advocacy tool for addressing the challenges immediately

Provide possible reasons for some findings e.g. disparity on samples collected in the facility

How can these results be used - can there be targeted training by cadre and by facility .

Limitations

- It is difficult to evaluate the stated limitation “ *Interviewed results were dependent on the laboratory personnel's response”* when the content of the questionnaire is not made clear in the methods
- The authors need to be clear which bias is inferred here – samples rejected is something extracted from records – are the authors implying the lab submitted wrong results -
- Can the authors articulate what mechanisms in place were put in place to ensure the data is objective ?

The Conclusion sounds vague

- the laboratory system require strengthening to ensure high-quality services and optimal resource utilization. It may be valuable to specify the gaps in pre analytical that needs to be addressed .

*Tables and figures – see comments above*
