# Supplementary material for: Pre-analytical errors during HIV viral load (HIV-VL) and early infant diagnosis (EID) testing in Cameroon
Source: PLoS One. 2026 Apr 27;21(4):e0324886. doi: 10.1371/journal.pone.0324886 (PMC13120008; doi:10.1371/journal.pone.0324886)
Supplement: Attachment — Submitted filename: Review Comments- AM 23 jan 2026.docx [file pone.0324886.s007.docx]

**Overall Comments**

Thank you again for the opportunity to review this important and timely manuscript. The paper has improved substantially since the previous version, and the key message is now clearer. However, further revisions are needed to improve clarity and conciseness. In particular, the manuscript would benefit from reducing repetition of similar points (e.g., description of the setting and laboratory network), tightening the methods section, and ensuring a clearer separation between methods, results, and discussion. Shortening and streamlining several sections will make the paper more direct, articulate, and impactful.

**Methods**

The sampling and data collection section of the Methods requires clarification and tightening. It remains unclear how the three data collection tools were used:

- the data abstraction tool,
- the self-administered questionnaire, and
- the specimen collection and transport checklists.

Please clarify:

- the specific purpose of each tool,
- the type of data collected by each,
- the sequence in which they were used,
- who completed each tool, and
- how the data were captured (e.g., which database was used and by whom).

This information is critical to understanding the study design and ensuring reproducibility.

**Results**

There is repeated description of the study setting (e.g., “17 laboratories”) across several sections, which risks confusing the reader and diluting key messages. Consider removing some or repetitive sentences, for example:

**Line 199-203:** some o the information is not discussed in the discussing section. Eg

“All specimens were collected from outpatients receiving HIV care and treatment services.” If not important consider leaving out – unless its linked to sample rejevction – are the authors suggesting there could be differences if other conditions are involved – which could be but if not discussed later, it leaves the result hanging ..

**Line 203:** There is a tendency to interpret findings rather than simply present results. For example, statements explaining *why* the Centre region had the largest proportion of specimens should be reserved for the Discussion section.

**Line 206–207:** The statement comparing rejection rates to national targets introduces interpretation and may also be moved to the Discussion or simply somewhere on the table.

**Line 253–254:**
The statement on uneven distribution of specimen rejection introduces analysis and interpretation. Consider presenting the findings descriptively here and moving interpretation to the Discussion.

**Lines 267–270:** again intepretatioin that could be used in discussion section instead of here.

**Line 279:**There is a reference to Table 2, but the table does not clearly present the specific error types mentioned in the paragraph (e.g., labeling errors). Ensure alignment between text and table content.

**Line 295:** Table 2 appears to be repeated in a different format. The same comments apply—consider removing redundancy.

**Table 3:** The information presented largely overlaps with Table 2. Please consider merging these tables or retaining only one to avoid duplication.

**Table 1:** The table key indicates that data were unavailable for the East, South, Adamawa, and West regions. However, similar symbols or dashes appear for other regions without explanation. Please clarify whether EID data were also unavailable for those regions and ensure consistent notation.

**Discussion**

The Discussion would be more impactful if it **began with the current second paragraph**, which clearly states the key message—namely, that specimen rejection rates exceed national thresholds and that targeted interventions are needed to improve sample collection, transport, and other pre-analytical processes.

Findings from **Table 2** indicate that distance from laboratories and high sample volumes are risk factors for specimen rejection. Please expand the discussion to explore **possible reasons** for these associations (e.g., transport delays, staff workload, cold-chain challenges).

When comparing rejection rates with other studies, the argument would be strengthened by discussing **why** rates may be similar or different—such as similarities in health system structure, patient populations, laboratory networks, or logistical constraints. If these factors are unknown, this should be stated.

**Conclusion**

**Line 369:**
The conclusion recommends validated sampling manuals, electronic test request forms, staff training, and regular on-site supervision. However, these interventions are not clearly discussed earlier. The Discussion should explicitly explain **how and why** these measures would reduce rejection rates. For example:

- Are sampling manuals absent or poorly adhered to?
- Are errors driven by lack of training, supervision, or system design?

Clarifying these points will strengthen the link between findings and recommendations.

**Other Comments**

**Line 43:**
Please clarify what is meant by “laboratory performance.” Is this referring to specimen rejection rates, poor specimen quality, or key performance indicators (KPIs)? These are related but distinct concepts.

**Line 62:**
Add “other information discrepancies” to clearly distinguish these from previously mentioned mismatched patient identification.

**Line 69:**
Clarify the message to emphasize that **sample collection and transport quality need strengthening**, highlighting the need for targeted interventions to reduce pre-analytical errors arising from poor sample management at health facilities responsible for HIV biological monitoring in Cameroon.

**Line 158:**
The paragraph on “reasons for sample rejection” appears to present results rather than methods. Since this was one of the research questions, consider moving this content to the Results section.

**Line 166:**
The program description paragraph contains background information that may be more appropriate for the Introduction (possibly at the end) or may already be covered in the Setting section. Consider relocating or removing it to avoid repetition.
