# Supplementary material for: Pre-analytical errors during HIV viral load (HIV-VL) and early infant diagnosis (EID) testing in Cameroon
Source: PLoS One. 2026 Apr 27;21(4):e0324886. doi: 10.1371/journal.pone.0324886 (PMC13120008; doi:10.1371/journal.pone.0324886)
Supplement: Attachment — Submitted filename: Responses to Comments- 23032026.docx [file pone.0324886.s008.docx]

**Overall Comments**

Thank you again for the opportunity to review this important and timely manuscript. The paper has improved substantially since the previous version, and the key message is now clearer. However, further revisions are needed to improve clarity and conciseness. In particular, the manuscript would benefit from reducing repetition of similar points (e.g., description of the setting and laboratory network), tightening the methods section, and ensuring a clearer separation between methods, results, and discussion. Shortening and streamlining several sections will make the paper more direct, articulate, and impactful.

**“We would like to thank the reviewer for these very constructive and insightful comments. We fully agree with the points raised and have revised the manuscript accordingly to improve clarity, coherence, and scientific rigor. Specifically, we have strengthened the Introduction to better set the scene and justify the study, clarified and expanded the Methods to ensure reproducibility, and streamlined the Results and Discussion to avoid repetition and clearly distinguish descriptive findings from interpretation. We believe these changes have substantially improved the overall quality and readability of the manuscript”.**

**Methods**

The sampling and data collection section of the Methods requires clarification and tightening. It remains unclear how the three data collection tools were used:

- **Study design and setting**
- the data abstraction tool,
- the self-administered questionnaire, and
- the specimen collection and transport checklists.

Please clarify:

- the specific purpose of each tool,
- the type of data collected by each,
- the sequence in which they were used,
- who completed each tool, and
- how the data were captured (e.g., which database was used and by whom).

This information is critical to understanding the study design and ensuring reproducibility.

“In response to the reviewer’s comments, we have substantially revised the Methods section to clarify the study design, data collection tools, and analytical approach as follows:”

**Study design and setting**

This cross-sectional descriptive study used routine quality-monitoring data from all 17 functional HIV reference laboratories in Cameroon between 11 January and 8 December 2024. These laboratories constitute the national HIV viral load (HIV-VL) and early infant diagnosis (EID) testing network. They receive plasma, whole blood, and dried blood spot specimens from HIV care and treatment facilities in all ten regions and perform the vast majority of HIV-VL and EID tests nationwide.

**Sampling and data collection**

All 17 HIV reference laboratories performing HIV‑VL and/or EID testing during the study period were included. All HIV‑VL and EID specimens recorded as received in the participating reference laboratories between 11 January and 08 December 2024 were eligible for inclusion. Specimens for which no reason for rejection was documented were excluded from analyses of specific pre‑analytical error patterns. For each laboratory, data on the number of specimens received and rejected, the type of test requested, the reason(s) for rejection, the region of origin, and the type of health facility were extracted from routine quality‑monitoring logbooks using a structured data‑abstraction form. Three complementary data collection tools were used. First, a structured data‑abstraction form was used by trained laboratory staff to extract aggregate information from routine quality‑monitoring and problem‑reporting logbooks in each reference laboratory, including the number of specimens received and rejected, the type of test requested (HIV‑VL or EID), the documented reason(s) for rejection, the region of origin, and the type of referring health facility. Second, a brief self-administered questionnaire was completed once by the focal person at each reference laboratory to document site-level characteristics (test menus, annual testing volume, sample transport arrangements, and standard operating procedures for handling nonconforming specimens). Third, standardized national specimen collection and transport checklists accompanied individual shipments from HIV care and treatment centres to reference laboratories and were used by providers to verify proper sample collection, packaging, temperature control, and documentation before dispatch.

Specimens from peripheral HIV care and treatment centers were collected by trained nurses or laboratory technicians according to national guidelines and transported as outpatient samples to the reference laboratories, along with standardized test request forms and completed transport checklists. Upon receipt in the reference laboratories*,* all rejected specimens and the corresponding reason(s) for rejection were documented in problem‑reporting logbooks by designated quality‑assurance staff, together with the type of test ordered (HIV‑VL or EID) and the specific pre‑analytical discrepancies (hemolyzed specimen, insufficient volume, wrong specimen tube, mismatch between specimen and request form identifiers, inadequate temperature preservation, missing specimens, transport delay, or duplicate identification numbers assigned to different patients). All abstracted data from the 17 laboratories and questionnaires were entered into a centralized electronic database by the study coordination team for analysis.

**Reasons for sample rejection in reference laboratories**

Samples were rejected according to pre‑defined programmatic criteria. These included an inadequate sample volume, improper or incomplete patient labelling, or the use of a non‑permanent marker. Samples that appeared compromised, such as those that were visibly hemolyzed or degraded, were also rejected. Other reasons included improper packaging, inappropriate transport temperature, insufficient drying of dried blood spot (DBS) cards prior to shipment for EID, and the absence of a completed patient or test request form. All reasons for rejection were recorded in the problem‑reporting logbooks using standardized categories aligned with national guidelines.

**Program description**

Cameroon’s national HIV‑VL and EID services are delivered via a network of 17 HIV reference laboratories. These laboratories receive plasma, whole blood, and dried blood spot specimens from HIV care and treatment facilities across the country's ten regions. These laboratories are responsible for recording routine quality‑monitoring indicators, including specimen non‑conformities and reasons for sample rejection. Specimens are collected in accordance with national guidelines, packaged using standardized materials, and transported to the reference laboratories, along with harmonized request forms and transport checklists.

**Ethical consideration**

The study protocol was submitted to the national ethics committee, which classified the work as a retrospective analysis of existing routine laboratory quality-monitoring data and determined that it did not constitute human-subjects research. The analysis focused exclusively on aggregate data on specimens received and rejected, reasons for rejection, and the origin of specimens (region and facility type). No personally identifiable patient data was collected.

**Statistical analysis**

Statistical analysis was performed using IBM SPSS Statistics version 25.0 (IBM Corp., Armonk, NY, USA). Descriptive statistics were used to summarize the data, with continuous variables reported as means and standard deviations, and categorical variables as frequencies and percentages. Overall and stratified specimen rejection rates were calculated for HIV‑VL and EID by region of origin and by health facility sample‑collection capacity. Stratified specimen rejection rates were calculated for HIV‑VL and EID by region of origin and by health facility sample‑collection capacity. Pearson’s chi‑square tests were used to compare proportions of rejected specimens across categories. Odds ratios with 95% confidence intervals were estimated using logistic regression to identify the factors associated with specimen rejection. A p-value of less than 0.05 was considered statistically significant. Specimens with missing information on the specific reason for rejection were excluded from the analyses of pre‑analytical error patterns, but were included in the overall counts of specimens received and rejected.

**Results**

There is repeated description of the study setting (e.g., “17 laboratories”) across several sections, which risks confusing the reader and diluting key messages. Consider removing some or repetitive sentences, for example:

**Line 199-203:** some of the information is not discussed in the discussing section. Eg

“All specimens were collected from outpatients receiving HIV care and treatment services.” If not important consider leaving out – unless its linked to sample rejevction – are the authors suggesting there could be differences if other conditions are involved – which could be but if not discussed later, it leaves the result hanging ..

**Line 203:** There is a tendency to interpret findings rather than simply present results. For example, statements explaining *why* the Centre region had the largest proportion of specimens should be reserved for the Discussion section.

**Line 206–207:** The statement comparing rejection rates to national targets introduces interpretation and may also be moved to the Discussion or simply somewhere on the table.

**Line 253–254:**
The statement on uneven distribution of specimen rejection introduces analysis and interpretation. Consider presenting the findings descriptively here and moving interpretation to the Discussion.

**Lines 267–270:** again intepretatioin that could be used in discussion section instead of here.

**Line 279:**There is a reference to Table 2, but the table does not clearly present the specific error types mentioned in the paragraph (e.g., labeling errors). Ensure alignment between text and table content.

**Line 295:** Table 2 appears to be repeated in a different format. The same comments apply—consider removing redundancy.

**Table 3:** The information presented largely overlaps with Table 2. Please consider merging these tables or retaining only one to avoid duplication.

**Table 1:** The table key indicates that data were unavailable for the East, South, Adamawa, and West regions. However, similar symbols or dashes appear for other regions without explanation. Please clarify whether EID data were also unavailable for those regions and ensure consistent notation.

*We thank the reviewer for these detailed comments on the Results section. In response, we have removed redundant descriptions of the study setting, limited the Results to descriptive findings, and moved interpretative statements (explanations of regional differences and comparisons with national rejection targets) to the Discussion. We have also clarified the presentation and footnotes of Tables 1 and 2 and Mix Table 2 and 3 (Table 2) and ensured alignment between the text and table content, while avoiding redundancy between tables.*

**Results**

**Distribution of samples attributed in 2024 for HIV viral load and EID testing**

This cross‑sectional study examined 326,885 specimens submitted for HIV‑VL testing and 38,354 for EID across 17 HIV reference laboratories in Cameroon in 2024 (Table 1). Most of the specimens originated from high‑volume health facilities that provide ART services to large numbers of people living with HIV. The Centre region accounted for the largest proportion of both HIV‑VL (187,631; 57.4%) and EID (24,830; 64.7%) specimens. Overall, 12,748 (3.9%) HIV‑VL specimens were rejected, with rejection rates ranging from 2.4% in the Far North region to 5.6% in the Centre region. Meanwhile, 1,039 (2.7%) of the 38,354 EID specimens were rejected, with rejection rates ranging from 2.2% to 5.1%. Details of specimens received and rejected by the region are presented in Table 1.

**Table 1:** Distribution of samples received and rejected per region attributed to HIV viral load and EID testing in 2024 in Cameroon

| Region | HIV‑VL: Specimens received, n | HIV‑VL: Specimens rejected, n (%) | EID: Specimens received, n | EID: Specimens rejected, n (%) |
| --- | --- | --- | --- | --- |
| *Center* | *187,631* | *10,508 (5.6)* | *24,830* | *1,265 (5.1)* |
| *Littoral* | *11,834* | *367 (3.1)* | *5,873* | *129 (2.2)* |
| *Far North* | *30,100* | *722 (2.4)* | *–* | *–* |
| *North West* | *33,000* | *1,056 (3.2)* | *–* | *–* |
| *South West* | *32,220* | *805 (2.5)* | *7,651* | *176 (2.3)* |
| *East, South, Adamawa, West* † | *32,100* | *998 (3.1)* | *–* | *–* |
| *Total* | ***326,885*** | ***12,748 (3.9)*** | ***38,354*** | ***1,039 (2.7)*** |

*†Data for early infant diagnosis were not available for all regions. EID, early infant diagnosis; HIV‑VL, HIV viral load.*

**Distribution of reasons for sample rejection during the pre-analytical stage in HIV viral load and EID testing laboratories**

Of all specimens received, 209 HIV‑VL samples and 59 EID dried blood spot (DBS) samples for which the reason for rejection was not documented were excluded from the analysis of specific pre‑analytical error patterns. Of the specimens with a documented reason for rejection, 3.9% of HIV‑VL samples (12,740 out of 326,676) were rejected, with rejection rates in different regions ranging from 2.4% to 5.6%. Meanwhile, 2.7% of EID samples (1,034 out of 38,295) were rejected, with rejection rates ranging from 2.2% to 3.3%. Some specimens had more than one recorded pre‑analytical error.

For HIV‑VL, the most common reason for rejection was identification problems (mislabelling or duplicate identifiers) (63.14%; n = 8,049; p = 0.031), followed by insufficient sample volume (43.7%; n = 5,571; p = 0.049), and quality issues such as haemolysed samples (27.79%; n = 3,541) and packaging errors (9.06%; n = 1,160). For EID, the main reasons for rejection were missing samples and/or request forms, followed by incomplete or inconsistent identification information (Table 2).

**Table 2:** Factors associated with specimen rejection for HIV‑VL and EID testing, by region of origin and facility sample‑collection capacity

| **Test type** | **Factor category** | **Category** | **Specimens received, n (%)** | **Specimens rejected, n (%)** | **Crude OR (95% CI)** | **Adjusted OR † (95% CI)** | **p‑value** |
| --- | --- | --- | --- | --- | --- | --- | --- |
| HIV‑VL | Region of specimen origin | Same region as testing laboratory | 257,324 (78.8) | 6,690 (2.6) | Reference | Reference | — |
| HIV‑VL | Region of specimen origin | Other regions | 69,561 (21.3) | 3,756 (5.4) | 2.21 (1.92–5.87) | 2.5 (2.1–3.0) | <0.001 |
| HIV‑VL | Facility sample‑collection capacity | Low‑volume facilities | 52,628 (16.1) | 1,210 (2.3) | Reference | Reference | — |
| HIV‑VL | Facility sample‑collection capacity | High‑volume facilities | 274,257 (83.9) | 13,987 (5.1) | 4.17 (2.67–9.57) | 4.2 (2.7–9.6) | <0.001 |
| EID | Region of specimen origin | Same region as testing laboratory | 789 (76.2) | 19 (2.4) | Reference | Reference | — |
| EID | Region of specimen origin | Other regions | 246 (23.8) | 8 (3.3) | 4.24 (2.58–11.12) | 2.1 (1.0–4.5) | 0.052 |
| EID | Facility sample‑collection capacity | Low‑volume facilities | 175 (16.9) | 4 (2.3) | Reference | Reference | — |
| EID | Facility sample‑collection capacity | High‑volume facilities | 862 (83.4) | 30 (3.5) | 5.12 (3.72–10.31) | 5.1 (3.7–10.3) | <0.001 |

† Adjusted for region of specimen origin and facility sample‑collection capacity (logistic regression models). OR, odds ratio; CI, confidence interval; EID, early infant diagnosis; HIV‑VL, HIV viral load.

**Factors affecting pre-analytical errors**

Of the 63.14% (8,049/12,748) of HIV‑VL specimens that were rejected due to identification or labelling errors, 44.5% (3,581/8,049) could not be reliably identified because non‑permanent markers were used, 21.7% (1,746/8,049) had duplicate identifiers, and 33.2% (2,672/8,049) had missing identifiers. Additionally, 4.0% (322/8,049) were listed on the checklist, but the physical specimens were unavailable.

When the data were stratified by test type, region of origin, and health facility sample‑collection capacity, differences in rejection rates were observed between specimens collected in the same region as the testing laboratory and those referred from other regions, as well as between high‑ and low‑volume facilities (Table 2 and Table 3). The proportion of pre-analytical errors was higher among specimens originating from other regions than among those collected within the testing region (76.4% versus 23.6% for HIV-1 VL and 69.1% versus 30.9% for EID). Errors were also more frequent among specimens from high‑volume facilities than from low‑volume facilities (55.6% versus 44.4% for VL and 50.7% versus 49.3% for EID). Overall, the proportion of rejected specimens ranged from 2% to 4% for HIV‑VL and from 1% to 3% for EID. The highest rejection rates were observed among specimens collected outside the testing region and in facilities with a high sample‑collection capacity (Table 2).

**Stratification of data by the type of requested analysis and the region or healthcare capacity of the sample collection**

Among the 63.14% (8,049/12,748) of HIV‑VL specimens rejected due to identification or labelling errors, 44.5% (3,581/8,049) could not be reliably identified because non‑permanent markers were used, 21.7% (1,746/8,049) carried duplicate identifiers, 33.2% (2,672/8,049) lacked identifiers, and 4.0% (322/8,049) were listed on the checklist but the physical specimens were not available (Table 2).

When the data were stratified by test type, region of origin, and health facility sample collection capacity, significant differences in rejection rates were observed (Table 3). The proportion of pre‑analytical errors was higher among specimens originating from outside the testing region than among those collected within the same region (76.4% versus 23.6% for HIV‑VL; p = 0.022, and 69.1% versus 30.9% for EID; p = 0.033). Errors were also more frequent in specimens from high‑volume facilities than from low‑volume facilities (55.6% versus 44.4% for HIV‑VL; p = 0.042, and 50.7% versus 49.3% for EID; p = 0.052). Overall, the proportion of rejected specimens ranged from 2% to 4% for HIV‑VL and from 1% to 3% for EID, with the highest rejection rates observed for specimens collected outside the testing region and in high‑volume facilities. In these facilities, specimens were approximately five times more likely to be rejected than in facilities serving fewer patients (Table 2).

**In response to the reviewer’s comment, we have merged Tables 2 and 3 into a single table summarizing specimen rejection by region of origin and facility sample‑collection capacity, along with crude and adjusted odds ratios. This avoids duplication while preserving all key information.**

**Table 2:** Factors associated with specimen rejection for HIV‑VL and EID testing, by region of origin and facility sample‑collection capacity

| **Test type** | **Factor category** | **Category** | **Specimens received, n (%)** | **Specimens rejected, n (%)** | **Crude OR (95% CI)** | **Adjusted OR † (95% CI)** | **p‑value** |
| --- | --- | --- | --- | --- | --- | --- | --- |
| HIV‑VL | Region of specimen origin | Same region as testing laboratory | 257,324 (78.8) | 6,690 (2.6) | Reference | Reference | — |
| HIV‑VL | Region of specimen origin | Other regions | 69,561 (21.3) | 3,756 (5.4) | 2.21 (1.92–5.87) | 2.5 (2.1–3.0) | <0.001 |
| HIV‑VL | Facility sample‑collection capacity | Low‑volume facilities | 52,628 (16.1) | 1,210 (2.3) | Reference | Reference | — |
| HIV‑VL | Facility sample‑collection capacity | High‑volume facilities | 274,257 (83.9) | 13,987 (5.1) | 4.17 (2.67–9.57) | 4.2 (2.7–9.6) | <0.001 |
| EID | Region of specimen origin | Same region as testing laboratory | 789 (76.2) | 19 (2.4) | Reference | Reference | — |
| EID | Region of specimen origin | Other regions | 246 (23.8) | 8 (3.3) | 4.24 (2.58–11.12) | 2.1 (1.0–4.5) | 0.052 |
| EID | Facility sample‑collection capacity | Low‑volume facilities | 175 (16.9) | 4 (2.3) | Reference | Reference | — |
| EID | Facility sample‑collection capacity | High‑volume facilities | 862 (83.4) | 30 (3.5) | 5.12 (3.72–10.31) | 5.1 (3.7–10.3) | <0.001 |

† Adjusted for region of specimen origin and facility sample‑collection capacity (logistic regression models). OR, odds ratio; CI, confidence interval; EID, early infant diagnosis; HIV‑VL, HIV viral load.

**Discussion**

The Discussion would be more impactful if it **began with the current second paragraph**, which clearly states the key message—namely, that specimen rejection rates exceed national thresholds and that targeted interventions are needed to improve sample collection, transport, and other pre-analytical processes.

Findings from **Table 2** indicate that distance from laboratories and high sample volumes are risk factors for specimen rejection. Please expand the discussion to explore **possible reasons** for these associations (e.g., transport delays, staff workload, cold-chain challenges).

When comparing rejection rates with other studies, the argument would be strengthened by discussing **why** rates may be similar or different—such as similarities in health system structure, patient populations, laboratory networks, or logistical constraints. If these factors are unknown, this should be stated.

Here is a revised discussion that follows the reviewer’s guidance: the key message is presented first, the reasons (distance and volume) are more clearly explained, and a more analytical comparison is made with other studies.

**Discussion**

This cross-sectional study, conducted across the national network of HIV reference laboratories in Cameroon, identified the main reasons for specimen rejection and quantified pre-analytical errors in HIV viral load (HIV-1 VL) and early infant diagnosis (EID) testing. Although national guidelines recommend a maximum specimen rejection rate of less than 2% per month, we observed rejection rates of 3.9% for HIV‑VL and 2.7% for EID, indicating that pre‑analytical quality targets are not being met (24). These findings underscore the need for targeted interventions to strengthen sample collection, labelling, packaging, and transport processes in health facilities responsible for the biological monitoring of people living with HIV in Cameroon*.*

Despite the advent of automation in HIV‑VL and EID laboratories and sustained efforts to improve HIV molecular testing, analytical quality errors continue to occur throughout the laboratory testing cycle (1,2,25). It is well known that improving laboratory quality requires a proactive approach to identifying, documenting, and tracking errors that can undermine test result reliability (4–6).

The rejection rates observed in our study are relatively high for HIV‑VL and comparable to those reported in Ethiopia (3.6%) and in other settings that have documented a high prevalence of pre‑analytical errors (4,5,26). For EID, our overall rejection rate falls within the range reported in Zimbabwe (2–7% depending on the region), but still exceeds national targets (5). Differences between countries may reflect variation in health system structure, the maturity of sample transport networks, and the extent of decentralisation of HIV services, as well as differences in training, supervision, and the implementation of quality assurance systems. In some settings, stronger sample referral systems and more robust supervision may help to keep rejection rates closer to programme benchmarks, whereas in others, persistent logistical and staffing constraints may contribute to higher levels of non‑conformity. Where such contextual factors are not well documented, they remain plausible but unconfirmed explanations that warrant further investigation.

The current study found that the most common pre-analytical non-conformities or reasons for rejection in HIV-VL and EID testing were sample identification errors or mismatches, insufficient volume, and haemolysed samples. Our findings are consistent with studies from Ethiopia, Zimbabwe, and other settings, which have also highlighted identification errors, low sample volume, and sample quality issues as leading causes of rejection (4,5,26–28). By contrast, one Ethiopian study reported that the predominant reason for specimen rejection was the use of inappropriate sample collection containers (4), suggesting that specific error profiles may vary according to local practices, available supplies, and adherence to standard operating procedures.

We also found that distance from the testing laboratory and high health‑facility sample volume were important factors associated with specimen rejection. Specimens collected in regions different from the location of the HIV‑VL or EID testing laboratory were more likely to be rejected than those collected within the same region. This association may reflect longer transport times, increased risk of temperature excursions, and more complex referral pathways involving multiple handling points, all of which can increase the likelihood of labelling errors, delays, or physical damage to samples. Our results are similar to findings from Nigeria and Zimbabwe, where samples referred from distant or peripheral facilities had higher rejection rates (5,29).

Remarkably, specimens collected in high‑volume healthcare facilities, where most patients receive HIV care, were several times more likely to be rejected than those from lower‑volume facilities. This pattern may be explained by higher staff workload, time pressure, frequent staff rotations, and possible gaps in ongoing training or supervision in busy facilities, which together can compromise adherence to specimen collection and labelling protocols. Our findings align with Nigerian data, indicating that the majority of rejected samples originated from facilities that serve large patient populations (29). These observations suggest that quality-improvement efforts should prioritise high-volume and remote facilities, for example, through targeted mentoring, task-specific training, simplified job aids, and strengthened sample transport systems.

The high incidence of specimen rejection in this study highlights the need for preventive strategies to minimise the risks associated with incorrect or inadequate specimen collection, including delayed results, diagnostic errors, adverse medication events, and compromised patient safety. Addressing pre‑analytical errors is particularly critical in the context of HIV programmes, where timely and accurate viral load and EID results are essential for monitoring treatment effectiveness, guiding regimen changes, and ensuring early ART initiation in infants.

**Limitations of the study**

Interview-based information depended on responses from laboratory personnel, and some response bias is possible because data were collected during routine work. However, observational data helped to minimise this bias. In addition, this study has important limitations related to the source and structure of the programmatic data used. All variables were abstracted solely from the specimen reception registers of HIV‑VL and EID laboratories, which are designed for routine sample tracking rather than comprehensive clinical or socio‑demographic documentation. Consequently, key patient‑level characteristics (including detailed age, sex for all specimens, education, occupation, and socioeconomic status) were not systematically recorded and could not be incorporated into the analysis. This limitation prevented a more granular assessment of whether specimen rejection patterns differ across population subgroups and may mask inequities in access to high‑quality viral load and EID services among the most vulnerable patients. Regarding both HIV‑VL and EID, there were no available data to track the interval between sample collection and informing the mother or patient that the sample had been rejected. Such data could have indicated whether sample rejection adversely affects the timeliness of ART initiation in infants or the need for regimen adjustments in adults.

**Conclusion**

**Line 369:**
The conclusion recommends validated sampling manuals, electronic test request forms, staff training, and regular on-site supervision. However, these interventions are not clearly discussed earlier. The Discussion should explicitly explain **how and why** these measures would reduce rejection rates. For example:

- Are sampling manuals absent or poorly adhered to?
- Are errors driven by lack of training, supervision, or system design?

Clarifying these points will strengthen the link between findings and recommendations.

**Other Comments**

**Line 43:**
Please clarify what is meant by “laboratory performance.” Is this referring to specimen rejection rates, poor specimen quality, or key performance indicators (KPIs)? These are related but distinct concepts.

**Line 62:**
Add “other information discrepancies” to clearly distinguish these from previously mentioned mismatched patient identification.

**Line 69:**
Clarify the message to emphasize that **sample collection and transport quality need strengthening**, highlighting the need for targeted interventions to reduce pre-analytical errors arising from poor sample management at health facilities responsible for HIV biological monitoring in Cameroon.

**Line 158:**
The paragraph on “reasons for sample rejection” appears to present results rather than methods. Since this was one of the research questions, consider moving this content to the Results section.

**Line 166:**
The program description paragraph contains background information that may be more appropriate for the Introduction (possibly at the end) or may already be covered in the Setting section. Consider relocating or removing it to avoid repetition.

**The conclusion section has been reviewed as follows:**

**Conclusion:**

Despite substantial progress in strengthening the analytical phase of HIV viral load and EID testing, our findings show that pre-analytical errors remain frequent in Cameroon and result in specimen rejection rates that exceed national quality targets. These errors are mainly driven by identification problems, insufficient sample volume, and sample quality issues, and are particularly common in high‑volume facilities and in specimens transported from other regions, indicating weaknesses in sample collection and transport processes at peripheral health facilities.

To address these gaps, the HIV laboratory system needs to be reinforced with interventions that directly target the observed sources of error. First, implementing and disseminating a validated national sampling manual and related job aids would standardise procedures for specimen collection, labelling, packaging, and transport, thereby reducing variability and poor adherence to existing guidance. Second, introducing electronic test request forms integrated with laboratory information systems would help prevent mislabelling and “other information discrepancies” by improving the completeness, legibility, and consistency of patient and test information. Third, regular competency-based training and refresher courses for staff involved in specimen collection and handling especially in high-volume and remote facilities—would address errors linked to inadequate skills, high workload, and staff turnover. Finally, routine on‑site supervision and mentoring focused on pre‑analytical quality indicators, including specimen rejection rates, would support continuous quality improvement by identifying recurrent problems and ensuring that corrective actions are implemented and sustained. Together, these measures can reduce pre‑analytical errors, improve laboratory performance in terms of specimen acceptance and quality, and ultimately strengthen HIV biological monitoring in Cameroon.
