# Supplementary material for: Pre-analytical errors during HIV viral load (HIV-VL) and early infant diagnosis (EID) testing in Cameroon
Source: PLoS One. 2026 Apr 27;21(4):e0324886. doi: 10.1371/journal.pone.0324886 (PMC13120008; doi:10.1371/journal.pone.0324886)
Supplement: Attachment — Submitted filename: Responses_Journal Requirements_09 April 2026.docx [file pone.0324886.s010.docx]

Journal Requirements:

1. If the reviewer comments include a recommendation to cite specific previously published works, please review and evaluate these publications to determine whether they are relevant and should be cited. There is no requirement to cite these works unless the editor has indicated otherwise. 

2. Please review your reference list to ensure that it is complete and correct. If you have cited papers that have been retracted, please include the rationale for doing so in the manuscript text, or remove these references and replace them with relevant current references. Any changes to the reference list should be mentioned in the rebuttal letter that accompanies your revised manuscript. If you need to cite a retracted article, indicate the article’s retracted status in the References list and also include a citation and full reference for the retraction notice.

Additional Editor Comments:

Dear authors,

I have read your revised manuscript with ID PONE-D-25-23115R2. However, I would suggest that you recheck the manuscript and revise it based on the following observations before it can be reconsidered for publication.

1. The title is too long and redundant. I would recommend a clear, and straightforward title. For example: We choose the proposition (a)

**(a) Pre-analytical errors in HIV viral load and early infant diagnosis testing in Cameroon**

(b) Pre-analytical errors and sample rejection in HIV viral load and early infant diagnosis testing in Cameroon

2. In Table 1, the total EID rejected is 1,039 (2.7%). The table row for the Centre region shows that the EID rejected is 1,265 (5.1%). Please reconcile the EID rejection counts in Table 1, because the Centre regional count exceeds the reported national total.

**We thank the reviewer for pointing out this inconsistency in Table 1. After verification of our dataset, we identified a typographical error in the row corresponding to the Center region. The correct number of EID rejected for the Centre region is 734, corresponding to 2.9%, and not 1,265 (5.1%) as initially reported. We have corrected Table 1 accordingly in the revised manuscript, and the regional values now sum to the national total of 1,039 (2.7%).**

3. From the previous revision, Table 2 and Table 3 were merged. The text still references both tables (L252).

We thank the reviewer for highlighting this oversight. We have now corrected this sentence so that it refers only to “Table 2” at line 252 of the revised version.

4. Statistical reporting in the manuscript should be improved. The authors, could as for example, report the exact p-values OR simply significance labels. In the current draft, both are being used. Please avoid the use of ‘NS’.

We thank the reviewer for this helpful comment regarding statistical reporting. In the revised manuscript, we have harmonized the reporting of statistical results throughout the text and tables.

5. In Table 2, the OR ranges of adjusted (2.7–9.6) appear to be a rounding of the crude estimate (2.67–9.57), which masks the differences. In Table 4 of the supplementary materials, the crude OR is 2.8 with 95% CI of 1.9–2.8. I recommend that where adjustments had minimal impact, it should be briefly mentioned in the text for clarity.

We thank the reviewer for this helpful comment and we agree with the concern raised. In the revised version of Table 2 and the corresponding supplementary Table 4, we now report crude and adjusted odds ratios and their 95% confidence intervals using a consistent number of decimal places, to avoid masking small differences between crude and adjusted estimates. We have also added a sentence in the Results section explicitly noting that, for high‑volume facilities and other key factors, adjustment had only a limited impact on the magnitude of the odds ratios. We add this sentence in result section Lines 233 to 235: “I**n multivariable analysis, the adjusted odds ratios for high‑volume facilities remained close to the crude estimates, indicating that adjustment had only a limited impact on these associations”**

6. Table 1 in the supplementary files is the same as Table 1 in the main text. The same is nearly true for Table 4 (Supplementary files) and Table 2 of the main text. I recommend removing redundant tables, and labeling supplementary tables appropriately (say Table S1, S2, etc.) and citing them appropriately in the main text.

We thank the reviewer for this helpful observation. In the revised manuscript, we have removed redundant tables from the supplementary files when they duplicated tables presented in the main text. The remaining supplementary tables have been relabeled sequentially as Table S1, Table S2, Table S3, and Table S4, and we have updated all corresponding citations in the main text to ensure consistent and appropriate referencing of the supplementary material.

7. Some references are incorrect, duplicated or have wrong/incomplete bibliographic information: For example,

a) Reference 25 and 26 should be:

25. Zhang L, Hu ZD. Clinical applications of machine learning in pre-analytical, analytical and post-analytical phases of laboratory medicine: a narrative review. AME Med J 2022;7:37.

26. Misganaw AS, Worku M, Bashea C, Nigus M, Yoseph Y, et al. (2019) Pre Analytical Errors in the HIV Anti Retro Viral Therapy (ART) Laboratory of Teaching Referral Hospitals in Addis Ababa, Ethiopia. Int J Virol AIDS 6:057. DOI: 10.23937/2469-567X/1510057

b) References 7 and 21 are the same.

We thank the reviewer for pointing out these reference inaccuracies. References 21, 25 and 26 have been corrected in the revised manuscript.

8. Other suggestions are in the manuscript file attached (Review)

[Note: HTML markup is below. Please do not edit.]

Reviewers' comments:

Reviewer's Responses to Questions
